# Relationship between Arterial Hypertension with Cognitive Performance in Elderly. Systematic Review and Meta-Analysis

**DOI:** 10.3390/brainsci11111445

**Published:** 2021-10-29

**Authors:** José Miguel Sánchez-Nieto, Uriel Dagoberto Rivera-Sánchez, Víctor Manuel Mendoza-Núñez

**Affiliations:** Research Unit on Gerontology, FES Zaragoza, National Autonomou, University of Mexico, Mexico City 04510, Mexico; cheverego@hotmail.com (J.M.S.-N.); riveraurield@gmail.com (U.D.R.-S.)

**Keywords:** high blood pressure, aging cognitive, memory

## Abstract

Background: Previous systematic reviews report that arterial hypertension (AHT) is associated with lower performance in cognition in the elderly. However, some studies show that with higher blood pressure, a better cognitive performance is obtained. Objective: The aim of this study was to determine the relationship between AHT with cognitive performance in the elderly. Methods: the review involved a search on PubMed, Scopus and PsycINFO databases from January 1990 to March, 2020 to identify the relationship among AHT and cognitive performance in older people. Results: 1170 articles were identified, 136 complete papers were reviewed, a qualitative analysis of 26 studies and a quantitative analysis of eight studies were carried out. It was found that people with AHT have a lower performance in processing speed SMD = 0.40 (95% CI: 0.25, 0.54), working memory SMD = 0.28 (95% CI: 0.15, 0.41) in short-term memory and learning SMD = −0.27 (95% CI: −0.37, −0.17) and delayed recall SMD = −0.20 (95% CI: −0.35, −0.05). Only one study found that higher blood pressure was associated with better memory performance. Conclusion: Our results suggest that high blood pressure primarily affects processing speed, working memory, short-term memory and learning and delayed recall.

## 1. Introduction

Regular blood pressure is 120/80 mmHg. When it gets to 140/90 or more in a chronic manner, it is considered as high blood pressure or arterial hypertension (AHT) [1]. The American Heart Association and the American Heart College have proposed to reduce this diagnostic criterion to 130/80 mmHg [2]; however, the relevance for Latin America is still pending [3].

It is estimated that there are 1130 million people worldwide with AHT [1]. It is associated with other diseases like diabetes [4], cancer [5], psychosocial stress [6], dementia or cognitive impairment [7]. It has such relevance that AHT has been considered by a group of experts as the main modifiable factor from middle age on to prevent dementia or cognitive impairment [8].

Hypertension is associated with anatomical and physiological changes which harm the brain. AHT increases by atherosclerosis and stiffness in blood vessels. Atherosclerosis is the thickening of arteries produced by the accumulation of fat, cholesterol and other substances, thereby, decreasing the blood vessels’ internal diameter. Additionally, it favors clot formation or thrombogenesis. Such stiffness is triggered by the blood vessels endothelium’s hypertrophy, increasing collagen and fibronectin deposition. All the above explains why hypertension is one of the main factors associated to stroke and dementia [9,10]. Blood vessels’ rigidity is a predictor of vascular events [11] and it generates mini strokes in basal ganglia and white matter arterioles, therefore, causing the so-called small vessel disease [12,13]. In addition, AHT is a risk factor for atrial fibrillation, a condition that increases the probability of developing cognitive impairment [14].

Hypertension also causes a reduced blood flow to the brain or hypoperfusion, due to a problem with self-regulation and microvascular rarefaction. Self-regulation is the blood vessel capacity of keeping a relatively constant blood flow through the brain, despite blood pressure levels in the rest of the body [15]. Constant high blood pressure affects self-regulation, causing the brain to decrease its blood flow, and thus protecting it from high blood pressure damage but increasing risk of ischemic injury. Besides, loss of micro vessels causes microvascular rarefaction, a change in the arterial and venous system which also reduces blood flow and potential blood compensation in vascular insufficiency or exclusionary conditions [9,10].

Likewise, the blood brain barrier is affected by high blood pressure, since plasmatic protein extravasation conducts to vascular, perivascular inflammation and microvascular thrombosis. All this contributes to white matter damage through inflammatory mediators, both oxygen and nitrogen reactive [12,16].

Hemorrhagic heart attack, ischemia and small vessel disease are all associated with vascular dementia [17]. Furthermore, an association between systolic pressure (>160 mmHg) and Alzheimer’s disease has been found (hazard ratio 1.25, IC95% 1.06, 1.47) [18], possibly because vascular damage increases amyloid plaque formation and neurofibrillary tangles [12]. Similarly, the hypoperfusion caused by constant high blood pressure activates different Alzheimer associated mechanisms and cognitive impairment [19]. 

The decrease of blood pressure in patients with hypertension reduces the risk of having dementia or mild cognitive impairment. Nonetheless, such an effect over cognitive functioning is controversial [20], particularly after the age of 60 years old [21].

Cognitive aging is heterogeneous: while knowledge-related processes increase, new-information processes decrease [22,23]. The most affected processes are attention-related, mainly processing speed, work memory and cognitive inhibition, whereas episodic memory and reasoning are the less affected ones [24,25,26]. Similarly, it has been discovered that hypertension primarily affects processing speed, memory and flexibility [27,28], similar processes which occur during aging. 

In two previous systematic revisions, an association among blood pressure and cognitive functions has been found. For example, an increase in high blood pressure is related with lower cognitive performance. In addition, higher and lower levels of blood pressure relate with cognitive performance, with an U-shaped relationship. Meanwhile, some studies show that with higher blood pressure, a better cognitive performance is obtained [27,28].

On the other hand, Forte et al. (2020), carried out a systematic review with the aim of measuring the effects of blood pressure on cognitive performance in adults and older adults. In this sense, they analyzed fifty studies and found that higher blood pressure is associated with a higher risk of cognitive decline in the young adult population. In contrast, higher blood pressure was reported as a protective factor for cognitive performance in older people, which they called the “cardiovascular paradox” [29].

The differences found in the studies might have been the result of the preferred design method in each revision. For instance, Van den Berg et al. (2009), included 24 studies, but only 11 of these had a group control [27]. Other elements that varied in the selected studies were the cut-off points for systolic and diastolic blood pressure to establish the diagnosis of AHT. In this sense, some studies considered it as 140/90 mmHg, while others were 160/95 mmHg [27,28]. The test used to evaluate cognitive functions included a screening test [28]. 

Due to the above, we performed a systematic revision to determine the relationship between high blood pressure and cognitive performance in older people.

## 2. Materials and Methods

### 2.1. Research Strategies

The reported items for the systematic revisions and meta-analysis protocols (PRISMA) were used to perform this revision (for a detailed summary see Appendix A). The protocol has not been registered. We researched published articles from January 1990 to March 2020 through Pubmed, Scopus and PsycINFO data bases with the search key words: “hypertension” OR “blood pressure” AND “cognition” AND “older”.

### 2.2. Eligibility Criteria

For the studies to be included, they needed to fulfill the predefined requirements of PEO: Population, Exposition and Outcome. For this systematic revision we included the studies with the following designs: cross-sectional study, cohort study, case-control study; with a population between 50 and 80 years old; with one or more groups with hypertension and with a group or population without hypertension to assess the effect of AHT. Furthermore, the studies had to use at least two valid neuropsychological instruments to measure cognitive functions. We excluded systematic revision articles, case studies and basic research studies with animals. We also excluded studies with a population with a neurological or psychiatric disease (cerebrovascular event, dementia, cognitive impairment, depression, etc.) or some other disease affecting cognition in a significant manner like cancer, AIDS and renal insufficiency. Finally, we also excluded articles which only used a screening test to evaluate cognitive functions, for example, the Folstein Mental State mini test or the Montreal Cognitive Assessment.

### 2.3. Article Selection

An independent article revision was performed by the authors (JM S-N and UD R-S) at every stage, considering inclusion/exclusion criteria. We used Excel software to keep track of the revision process. When disagreement occurred among both authors, a third author (VM M-N) was involved to participate in the discussion. First, we eliminated repeated articles. Next was the selection of articles based on the title and abstract. Subsequently, the full articles were reviewed, and the articles were selected for qualitative synthesis and meta-analysis.

### 2.4. Analysis and Data Synthesis

The authors independently registered study type, sample characteristics (size, male/female ratio, age, scholarship and blood pressure, their hypertension definition and main results). Subsequently, we compared the compiled data and, in case of disagreement, the article was reviewed again.

The author JM S-N classified the tests and implemented tasks in each study, using the following categories: executive functions, work memory, processing speed, cognitive inhibition, short-term memory learning, delayed memory, and reasoning. We selected these processes because they are the ones most affected during aging [22,24].

The Newcastle-Ottawa Scale (NOS) was used to assess the quality case-control studies in the meta-analysis [30]. When the interventions and associated outcomes were assessed as sufficiently homogeneous and when sufficient information was available from the studies, quantitative data were pooled in the Review Manager (Version 5.3, The Cochrane Collaboration 2014) for meta-analysis. In this sense, in the event that the group with hypertension was divided by some characteristic, the scores were calculated to obtain only a mean score, standard deviation and sample size. The meta-analysis of these values was performed using the random effects model. The I^2^ statistic was used to assess inconsistencies between studies and describe the percentage of variability in effect. Heterogeneity was considered substantial if the I^2^ statistic was ≥50%. All effect sizes were calculated using standardized mean differences (SME), as all studies used a wide variety of scale measures.

## 3. Results

### 3.1. Studies Selection

**Figure 1:**
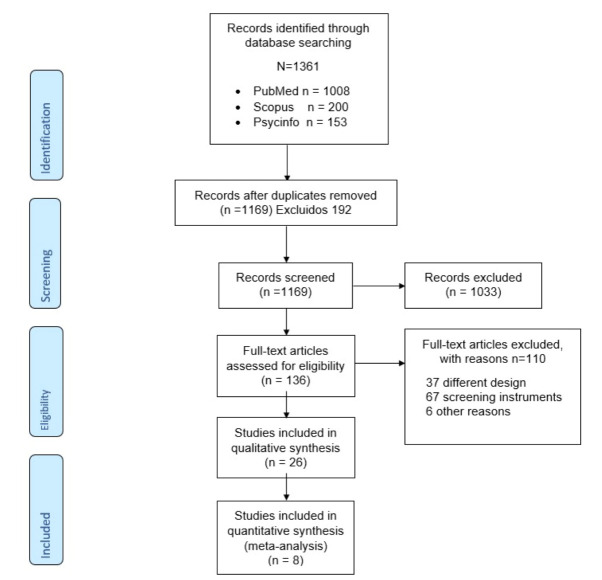
Study selection flow chart.

In the initial search strategy, we found 1169 articles, mainly on PubMed (After removing the duplicated publications), 1033 articles were rejected according in their titles and abstracts. 136 studies were reviewed, and out of these we excluded 37 due to differences regarding their methodology. Additionally, we excluded another 67 because those only used one screening instrument, and six were disqualified for other reasons. Upon completion, we ended up with 26 articles which met the eligibility criteria, of which 8 were included in the meta-analysis (Figure 1).

### 3.2. Studies Characteristics

We selected 26 studies, of which six were cross-sectional, nine were cohort and 11 were case-control (Table 1). The sample size of patients with hypertension ranged from 12 [31] to approximately 3200 [32]. The age of the participants was mostly around 70 years (Table 1). The percentage of men varied from 7% [33] to 69.4% [34]. Some studies perform a division by gender [35,36], and in five we did not find information on the composition by gender [37,38,39,40,41]. Most of the studies included participants with eight or more years of education (Table 1), in only two studies did most of the participants have less than eight years of education [42,43].

The definition of hypertension was by medical diagnosis or by being prescribed medications for high blood pressure (Table 1). In three of them we did not find the criteria to define arterial hypertension [41,44,45]. The cut-off points of systolic pressure to define hypertension varied in the studies, one of them was greater than 130 mmHg 3200 [46], five were greater than 140 mmHg [35,36,38,39,42], and seven studies were greater than 160 mmHg [32,40,43,47,48,49,50].

Eleven case-control studies were found without risk of bias and with adequate quality to perform a quantitative analysis (Appendix B). In some studies, they divided the sample with hypertension into two groups: controlled blood pressure (systolic pressure < 140 mmHg) or out of control [38,51], or into three groups: controlled blood pressure, out of control, and without treatment [39,42]

### 3.3. Relationship of Cognitive Performance with Arterial Hypertension

In most studies it is found that the higher the blood pressure or hypertension, the lower the cognitive performance. Only in one study was it found that higher the diastolic pressure contributed to a better cognitive performance [47]. In three studies it was found that a low or high blood pressure is related to a lower cognitive performance [32,45,51] and in two studies no relationship was found between hypertension and cognitive performance [52,53] (Table 1).

#### 3.3.1. Processing Speed

The processing speed was tested in 17 studies (Table 2). Seven studies found that higher blood pressure is associated with lower performance on processing speed tests [31,36,37,38,41,48,54]. Two studies found a relationship between lower performance in cognitive tests with lower diastolic pressure in people who do not receive treatment [51], and systolic in participants who suffered myocardial infarction [32].

**Figure 2:**
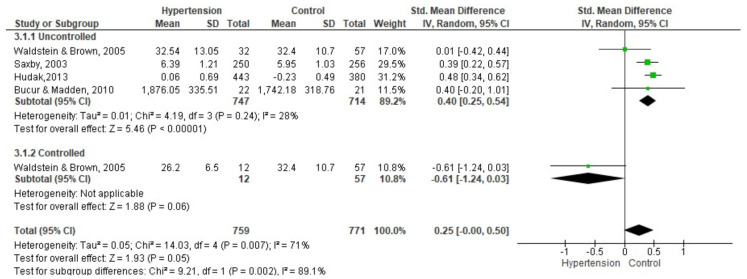
Meta-analysis on the comparison of groups with controlled and uncontrolled hypertension between the control group in processing speed. High score indicates lower performance.

Four studies with a total of 759 participants with hypertension and 771 controls were included in the processing speed meta-analysis (Figure 2). The results were analyzed from the digit and symbol substitution tests [37,46], trail making test A [31] and a measure composed of several tests [48]. In this indicator, a higher score indicates a lower cognitive performance. Two studies had subdivisions of the group with hypertension: one with several treatments [37], and another in untreated, uncontrolled and controlled [31], for which they were united, forming three groups; controlled hypertensive, uncontrolled hypertensive, and the control group.

The group with uncontrolled hypertension compared to the control group had a lower performance in processing speed SMD = 0.40 (95% CI: 0.25, 0.54; I2 = 28%; *p* = 0.24; n = 4 studies; hypertension, n = 747; control, n = 714). The group with controlled hypertension compared to the control group had a higher performance in processing speed SMD = −0.61 (95% CI: −1.24, 0.03; *p* = 0.06; n = 1 study; hypertension, n=12; control, n = 57).

#### 3.3.2. Working Memory

The working memory was evaluated in 13 studies, mainly with the Trail Making Test B and Digit Span Backwards (Table 2). Six studies found that higher blood pressure is associated with lower performance on working memory tests [31,36,37,38,40,48,51]. One study found that low diastolic pressure is associated with lower performance on working memory tests, particularly in the low-educated population [51]. 

**Figure 3:**
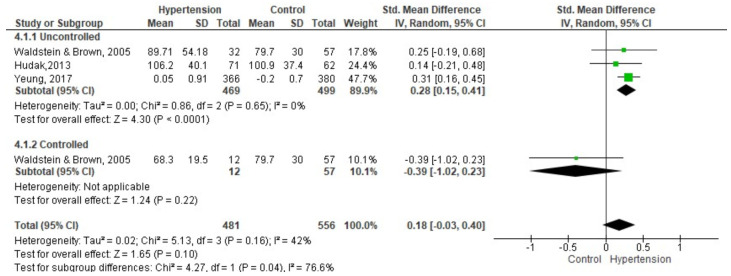
Meta-analysis on the comparison of groups with controlled and uncontrolled hypertension between the control group in working memory. High score indicates lower performance.

Three studies with a total of 481 participants with hypertension and 499 without hypertension were included in the meta-analysis of working memory (Figure 3). The results used were from the Trail Making Test B [31,37,52]. In this indicator, a higher score indicates a lower performance. The studies divided the groups into ingesting different drugs [37] and untreated, uncontrolled and controlled [31], for which they were united forming three groups: uncontrolled hypertension, controlled hypertension, and the control group.

The group with uncontrolled hypertension compared to the control group had a lower performance in working memory SMD = 0.28 (95% CI: 0.15, 0.41; I2 = 0%; *p* = 0.65; n = 3 studies; n hypertension = 469; n control = 499). The group with controlled hypertension compared to the control group had a better performance SMD = −0.39 (95% CI: −1.02, 0.23; *p* = 0.1 n = 1 studies; n hypertension = 12; n control = 57), but it is not statistically significant.

#### 3.3.3. Short-Term Memory and Learning

Short-term memory and learning were included in 23 studies. In most cases, short-term memory was measured with a word-learning task; however, the tests were different among the studies, because the number of words ranged from 10 to 20 (Table 2). There were differences in 11 studies [31,35,38,41,42,43,44,47,48,51,55]. On the other hand, 10 showed no differences [33,34,36,37,39,48,49,52,53,56]. Only one case showed a difference in visual memory [36]; meanwhile one study found a U-shaped relationship [32].

**Figure 4:**
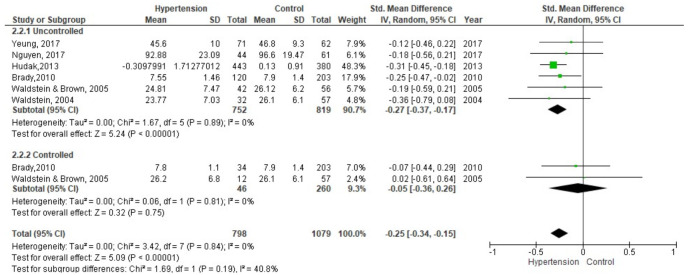
Meta-analysis on the comparison of groups with controlled and uncontrolled hypertension between the control group in short-term memory y learning.

Six studies with a total of 798 participants with hypertension and 819 without hypertension were included in the meta-analysis of short-term memory and learning (Figure 4). The results used were the California Verbal Learning Test (CVLT) [52], the Selective Reminding Test (SRT) [55], the Consortium to Establish a Registry for Alzheimer’s Disease (CERAD) [39], the Hopkins Verbal Learning Test (HVLT) [37], and the Logical Memory test [31,36]. Several studies had subdivisions in the hypertension group: with and without subjective memory problems [55], ingesting different medications [37], in men and women [36], untreated, uncontrolled and controlled [31,39]; reason why they were united forming three groups; controlled hypertensive, uncontrolled hypertensive and control group.

The group with uncontrolled hypertension compared to the control group had a lower performance in short-term memory and learning SMD = −0.27 (95% CI: −0.37, −0.17; I2 = 0%; *p* = 0.89; n = 6 studies; n hypertension = 752; n control = 819). The group with controlled hypertension compared to the control group had a lower performance SMD = −0.05 (95% CI: −0.36, 0.26; I2 = 0%; *p* = 0.81; n = 2 studies; n hypertension = 46; n control = 260).

#### 3.3.4. Delayed Memory

Delayed memory was included in 15 studies, and the time variation was from 2 to 30 min (Table 2). In two, higher pressure was found to be related to lower performance in delayed memory [31,32]. In one study it was found that lower diastolic pressure is related to lower performance in delayed memory, particularly in people who have suffered a stroke [32]. A study found that higher pressure is related to better performance in delayed memory in men [47].

**Figure 5:**
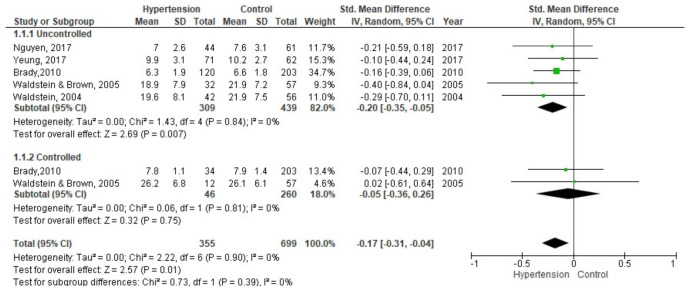
Meta-analysis on the comparison of groups with controlled and uncontrolled hypertension between the control group in delayed recall.

Five studies with a total of 355 participants with hypertension and 439 without hypertension were included in the delayed memory meta-analysis (Figure 5). The indicators used were CVLT delayed memory [52], SRT [55] CERAD [39], WISC, and logical memory [31,36]. Several studies had subdivisions in participants with hypertension: with and without subjective memory problems [55], in men and women [36], untreated, uncontrolled and controlled [31,39], for which they were united forming three groups: controlled hypertensive, uncontrolled hypertensive and control group.

The group with uncontrolled hypertension compared to the control group had a lower performance in memory delayed SMD = −0.20 (95% CI: −0.35, −0.05; I2 = 0%; *p* = 84; n = 5 studies; n hypertension = 309; n control = 439). The group with controlled hypertension compared to the control group had a lower performance SMD = −0.05 (95% CI: −0.36, 0.26; I2 = 0%; *p* = 0.81; n = 2 studies; n hypertension = 46; n control = 260).

#### 3.3.5. Other Cognitive Processes

Executive functions were assessed in 12 of the 26 studies (Table 2). In seven studies, higher blood pressure was found to be related to lower cognitive performance [34,35,42,48,51,52,56]. In one study, low and high diastolic pressure were found to be related to lower performance in executive functions [46].

Cognitive inhibition is only assessed in two studies using the Stroop interference task (Table 2). No significant difference was found between hypertensive and normotensive participants [31,52].

Reasoning was evaluated in seven studies (Table 2). In four studies, it was found that higher blood pressure is related to lower performance in reasoning tests [50,52,54,55].

## 4. Discussion

The present review was carried out with the objective of determining the relationship between high blood pressure and cognitive performance in older adults. The tests used were grouped into cognitive processes that decline during aging [22,24]. It was found through a quantitative analysis that higher blood pressure affects processing speed, working memory, short-term memory, and delayed memory.

The observed results in a qualitative analysis suggest that elderly people with hypertension have a lower level of performance than old people without this condition. This applied to executive function (seven out of 12 studies), work memory (six out of 13 studies), processing speed (seven out of 17 studies), short-term memory (nine out of 23 studies) or delayed memory (two out of 15 studies) and reasoning (four out of 7 studies). Nonetheless, in cognitive inhibition there is a need to perform more studies to be able to suggest or rule out an effect.

Our results contrast with another systematic revision made by Van den Berg, et al. (2009) [27], who concluded that the most affected process in hypertension patients is memory. In their revision, they performed a quantitative analysis where two articles included mild cognitive impairment patients [57,58], a condition which mainly affects memory [59].

In this sense, our revision has two main differences with the one made by Van de Berg et al. (2009) [27]. We excluded studies which integrated patients with mild cognitive impairment and we divided memory in two categories: short-term and delayed memory. The first one is related with processes like attention, while delayed memory is related to storage, which is an indicator of the hippocampus’s integrity [60]. This division was relevant, because it allowed us to show that in most studies there is a difference among AHT patients and people without this disease.

Most of the studies found reported that AHT is related to lower cognitive performance; however, in two studies a paradoxical effect is found. One study reported that higher diastolic pressure in men is related to higher cognitive performance [47], the other that the group with controlled AHT had higher performance compared to the control group [31], which was corroborated in the meta-analysis carried out in our study. One explanation for the paradoxical effect may be due to increased perfusion in the brain, which benefits from thickening of the arteries [61]. Another possible explanation may be an effect of the medications. In a systematic review, it was found that angiotensin II receptor blockers can prevent cognitive deterioration in people with AHT [62], and that these could have a positive effect on cognition. However, to corroborate the above it is necessary to carry out more research in this regard.

In four studies it was found that people with lower diastolic blood pressure had a lower performance, mainly in working memory and processing speed [32,45,51], particularly in people with less education [51]. Also, a lower working memory performance was found in people with low systolic pressure, especially if they had a myocardial infarction [32].

This is consistent with studies that report that both high and low blood flow can cause damage to the brain. On the one hand, hypertension can cause cerebrovascular accidents and lesions in the white matter [63]. On the other hand, low blood pressure may be associated with ischemic injuries [64,65]. Furthermore, very low blood pressure has been associated with the presence of Alzheimer’s disease 60. Considering the above, it would be necessary to maintain homeostatic blood pressure to preserve cognitive functioning.

A limit point is used to diagnose AHT, which causes significant variation. The value most frequently used is a blood pressure higher than 140/90 mmHg [66]. Another higher value set is 160/95 mmHG [67] or lower at 130/80 mmHg [2]. The latter can also explain the AHT effect on cognitive functions and inconsistencies in the different studies. For example, in this present study we did not find a significant difference among people with AHT and patients without this condition. Regarding work memory and processing speed, in one article authors used systolic pressure > 160 mmHg as diagnostic criteria [42]. Consequently, people with lower blood pressure might have already been affected, which would nullify the differences. On the other hand, in a different study, AHT was diagnosed with a blood pressure of 130 mmHg [46]. Thus, there could be people with these blood pressure levels who are not experiencing any impact on their cognitive functions.

Another relevant criterion is the previous diagnosis. For instance, people with AHT who maintain a controlled blood pressure by taking medication like angiotensin II receptor blockers can experience a lower impact on cognitive functions [62]. However, it is necessary to include the blood pressure criteria because if only the previous diagnosis is considered, people with a systolic pressure > 140 mmHg could be included in the control group [57]. That being the case, it would be better to divide AHT patients in at least three groups: controlled, uncontrolled with treatment and uncontrolled without treatment, besides the control group. [68,69]

We found that systolic pressure is related with a lower cognitive performance. In this sense, similar findings have been pointed out and reported in reviews narrative and systematic [64,65]. Another relevant indicator could be the pulse pressure, which is the difference between systolic and diastolic pressure, as whichever is higher might negatively affect cognitive functions [38,42,52].

The instruments used to measure cognitive functions are quite relevant to measure the effect of AHT as well. The most used ones in which there was a significant difference were the Trail Making Test A and B [36,37,38] and the digit-symbol substitution test [37,46,52]. Meanwhile, other instruments showed no difference, the work memory digits task being one example [31,36,39].

The National Institute of Neurologic Disorders and the Canadian Stroke Network have proposed three different evaluation protocols with AHT patients; the differences lie within the time (60 min, 30 min and 5 min) in which the tests mentioned above are included [70]. It is likely that these tests are sensitive enough to detect changes caused by AHT without structural damage; however, more research needs to be done to verify this.

One of the most relevant characteristics in the sample is the education level. In our revision, only two studies had a population with less than eight years of education [42,43]. Education is the main factor associated with cognitive reserve; this term is defined as a person’s capacity that if brain damage occurs, they can live with an adequate cognitive performance [71]. Therefore, the effects caused by an alteration in blood pressure may be greater; [51] however, more research is needed on this.

Our revision presents certain limitations: we failed to include studies with people older than 80 years old, and we did not revise grey literature nor the previously registered protocols. It would be advisable to carry out more studies analyzing other variables such as age, sex, follow-up time and academic level, comorbidities (atrial fibrillation and other arrhythmias, diabetes mellitus), medications and lifestyle (exercise, nutrition, sleep, smoking, and alcohol intake).

## 5. Conclusions

Our findings suggest that older people with AHT present a lower performance than old people without this condition, mainly in terms of processing speed, work memory, and memory. However, more studies with AHT patients need to be done, in both controlled and uncontrolled treatments. Furthermore, instruments sensitive to cognitive functions caused by AHT changes are essential in these kinds of studies, as is the capacity to identify the right AHT limit point (systolic or heart rate) where a change in cognitive functions is presented.

## Figures and Tables

**Table 1:** Description of studies on blood pressure and cognitive functions in older adults.

Study	Design (Years)	Goup	N	Age M (SD)	Sex (% Men)	Edu M (SD)	SBP M (SD)	DBP M (SD)	AHT Definition	Links Cognitive Impairment
Kritz-Silverstein et al., 2017 [40]	C-s	Man Hipertensive = 62.6%	693	73.8 (9.9)		Some college o more 77%	135 (20)	77.5 (9)	A SBP ≥ 160 DBP ≥ 90	Positive HTA
		Woman Hipertensive = 63.4	1022	73.2 (9.3)		Some college o more 62%	136 (21)	75 (9)		
Fischer et al. 2016 [34]	C-s	Hipertensive = 37.6%	85	71.4 (5.5)	69.4	14.3 (2.3)	130 (15)	74 (9)	A	Positive PP
Cherbuin et al., 2015 [47]	C (8)	Hipertensive = 51%	266	70.4 (1.4)	54	14.2 (2.6)	150 (19)	81 (10)	DM SBP ≥ 160 DBP ≥ 90	Women Positive SBD/ Men Negative DBP
Okusaga et al., 2013 [44]	C (5)	-	2312	61.7 (6.5)	26.9	-	147 (21)	84 (11)	-	Positive SBP
Dregan et al. 2013 [43]	C (8)	Hipertensive = 15%	5936	66.9 (10.1)	45	46% > 8	136 (19)	75 (11)	Normal < 140/90 Border 140–160/90–99 High 160/100	Positive SBP & DBP
Nation et al., 2010 [56]	C-s	Hipertensive = 52%	109	74.2 (10)	44	16.2 (2.3)	126 (13)	73 (9)	DM	Positive PP
Whitfield et al., 2008 [41]	C-s	-	361	61.5 (9.4)	-	12 (3.9)	141 (22)	82 (12)	-	Positive SBP
Singh-Manoux, et al., 2005 [35]	C (12)	Man	4158	43.9 (6.0)		70% > 8	Low = 45% Medium = 40% Hihg = 15%		Low < 120/80 Medium 120–139/80–89 Hihg > 139/90	Positive SBP & DBP
		Woman	1680	44.4 (6.0)		48% > 8	Low = 50% Medium = 34% High = 16%			
Robbins et al., 2005 [54]	C (23)	African HTA = 42%	147	54.7 (15.1)	61.2	12.6 (2.5)	139 (30)	84 (20)	DM	Positive SBP
		Caucasico Hipertensive = 36%	1416	56.6 (17.1)	56.2	14.4 (2.6)	138 (27)	81 (17.8)		
Waldstein, Giggey et al., 2005 [51]	C (11)	Hipertensive = 33.4%	847	70.6 (8.5)	59	16.6 (2.7)	139 (20)	82 (10.9)	A	Positive SBP U curve DBP
Hebert et al., 2004 [45]	C (6)	-	4284	74 (6.4)	38	12 (3.7)	140 (20)	77 (11.5)	-	U curve DBP
Ellias et al. 2004 [50]	C (20)	<47 years Hipertensive = 55.8%	285	34.9 (7.6)	48.4	14.3 (2.3)	130 (18)	80 (11.4)	SBP ≥ 160 DBP ≥ 90	Positive SBP
		≥47 years Hipertensive = 73.8%	244	58.1 (8.4)	48.8	14.2 (2.7)	146 (22)	83 (12.2)		
Steward et al., 2003 [53]	C (1.5)	Hipertensive = 58%	216	64 (5.3)	44	66% > 8	138 (28)	81 (18.1)	A	No association
Izquierdo-Porrera & Waldstein, 2002 [33]	C-s	Hipertensive = 53%	43	59 (11.2)	7	14 (2.5)	136 (21)	78 (11)	DM	Positive DBP
Morris et al., 2002 [32]	C-s	Hipertensive = 55%	5816	65 a 74 (59%) 75–84 (30%) >85(11%)	39	81% > 8	65–74 = 139 75–84 = 140 >85=138	65–74 = 79 75–84 = 75 >85=73	SBP ≥ 160 DBP ≥ 90	Positive SBP U curve DBP
Wei et al., 2018 [42]	C-c	Controlled hipertensive	695	61(19)	32.3	32% > 6	126 (10)	74 (8)	DMS BP ≥ 140 DBP ≥ 90	Positive AHT
		Treated high BP hipertensive	970	63 (9)	31.1	26.4% > 6	156 (19)	88 (14)		
		Untreated hipertensive	343	61 (9)	41.7	30% > 6 años	155 (19)	89 (13)		
		Normotensive	4724	58 (9)	34.3	32% > 6	118 (12)	71 (9)		
Yeung et al.,2017 [52]	C-c	Hipertensive	71	70.3 (6.5)	51	14.2 (2.5)	126 (10)	73 (8)	DM	No association
		Normotensive	62	70.2 (6.4)	49	15 (2.7)	119 (13)	71 (8)		
Nguyen et al., 2017 [55]	C-c	Hipertensive	44	79 (5)	46	17 (3.1)	149 (16)	83 (11)	DM	Positive AHT
		Normotensive	61	78.1 (5)	53	15.9 (2.7)	143 (17)	78 (8)		
Hudak et al., 2013 [37]	C-c	Hipertensive	390	73.3 (5.9)	-	13.9 (2.7)	-	-	DM	Positive AHT
		Normotensive	380	73.3 (5.9)	-	13.9 (2.7)	-	-		
Yasar et al., 2011 [38]	C-c (9)	Hipertensive SBP ≥ 160	190	74.1 (2.7)	-	93.6% ≥ 12	178 (12)	88 (17)	SBP > 140	Positive AHT
		Hipertensive SBP 140–159	113	73.7 (2.8)	-	91% ≥ 12	149 (5)	73 (12)		
		Normotensive	103	72.2 (2.9)	-	92.2% ≥ 12	129 (9)	67 (10)		
Bucur & Madden., 2010 [46]	C-c	Hipertensive	21	68 (4.7)	57	16.6 (4.7)	139 (7)	79 (9)	SBP ≥ 130 DBP ≥ 85	Positive AHT
		Normotensive	22	67.8 (5.1)	38	16.8 (2.3)	118 (8)	72 (5)		
Brady et al., 2005 [39]	C-c(2)	Controlled hipertensive	34	68.6 (6)	-	14.7 (2.4)	127 (9)	78 (8)	SBP ≥ 140 DBP ≥ 90	Positive AHT
		Treatable high BP hipertensives	45	69.5 (6.1)	-	14.5 (3.1)	153 (14)	89 (9)		
		Untreatable hipertensive	75	68.4 (7.5)	-	13.8 (2.6)	157 (16)	89 (11)		
		Normotensive	203	66 (7)	-	14.4 (2.6)	124 (9)	78 (6)		
Waldstein, Brown et al., 2005 [31]	C-c	Controlled hypertensive	12	68.4 (9.8)	69	16.8 (3.8)	133 (5)	76 (8)	DM	Positive AHT
		High BP Hypertensive	17	67.6 (5)	62	15.5 (3.2)	159 (9)	85 (6)		
		Normotensive	57	65.8 (6.5)	61	16.9 (2.7)	120 (11)	70 (7)		
		High BP No hypertensive	15	67 (6)	65	15.3 (2.7)	145 (8)	81 (5)		
Waldstein & Katzel, 2004 [36]	C-c	Hypertensive Man	31	68.9 (6.6)		16.5 (3.4)	147 (14)	80.4 (7.5)	SBP ≥ 140 DBP ≥ 90	Positive AHT
		Hypertensive Woman	11	66.1 (5.6)		14.6 (2)	146 (13)	81 (7)		
		Normotensive Man	30	66.8 (6.7)		16.9 (2.6)	123 (10)	72 (7)		
		Normotensive Woman	26	65.1 (6.6)		17 (2.8)	117 (11)	67 (7)		
Saxby et al., 2003 [48]	C-c	Hypertensive	250	74 (4)	47	10 (2)	165 (8)	89 (7)	SBP ≥ 160–79 DBP ≥ 90–99	Positive AHT
		Normotensive	256	74 (4)	56	10 (2)	131 (11)	74 (7)		
Harrington et al., 2000 [49]	C-c	Hypertensive	107	76 (4)	49	10 (2)	164 (9)	89 (7)	SBP ≥ 160 DBP ≥ 90	Positive AHT
		Normotensive	116	76 (4)	49	10 (2)	131 (10)	74 (7)		

M: Median; SD: standard deviation; EDU: Years Education; SBP: Systolic Blood Pressure; DBP: Diastolic Blood Pressure; PP: Pulse Pressure; AHT: Arterial hypertension; C-s: Cross-sectional study; C: Cohort study; C-c: Case-control study; BP; Blood Pressure; A: antihypertensive use; DM: Diagnosed by Medical.

**Table 2:** Cognitive processes included in the studies of blood pressure and cognitive performance in older adults.

Study	Executive Functions	Work Memory	Processing Speed	Cognitive Inhibition	Short-Term Memory and Learning	Differed Memory	Reasoning
Kritz-Silverstein et al., 2017 [40]	PVF SVF	TMTB *			WL (10) MV	MV (30 min)	
Fischer et al. 2016 [34]	DSB+ LNS + DEFS *				WL (16)	DWL (20 min)	
Cherbuin et al., 2015 [47]	PVF	DSB	SDMT TMTA TMTB		WL (16) ^P^	DLW (20 min) ^P^	
Okusaga et al., 2013 [44]	VF		DSS TMTA		WL (15) + WL (20 min) *		RM
Dregan et al. 2013 [43]	SVF + LCT				WL (10) + DWL *		
Nation et al., 2010 [56]	WCST + TMTB + VF D + VF *		TMTA		HM + DHM + WL + DWL		BD
Whitfield et al., 2008 [41]		AF	DSS *		HM **	HM (10 min)	
Singh-Manoux, et al., 2005 [35]	PVF * SVF *				WL (20)		MR
Robbins et al., 2005 [54]		DSB	DSS *				BD * ST *
Waldstein, Giggey et al., 2005 [51]	PVF SVF *	DSB TMTB^u^	TMTA^u^		WL (16) + DWL * VM + DVM *		
Hebert et al., 2004 [45]	SDMT + MMSE + EBMT^u^						-
Ellias et al. 2004 [50]			DSS		Ar + DSB + DSF		PC + PA + BD + OA *
Steward et al., 2003 [53]			TMT A		WL (10)	DWL (2 min)	
Izquierdo-Porrera & Waldstein, 2002 [33]		DSB			WL (10)	DWL (7 min)	CLOX
Morris et al., 2002 [32]			SDMT^u^		EBMT^u^	EBMT^u^	
Wei et al., 2018 [42]	TO + R7 + IC *				WL (10) + DWL (4 min) *		
Yeung et al.,2017 [52]	PVF SVF *	TMTB	DSS	SCW	WL (16)	DWL (20 min)	EPS *
Nguyen et al., 2017 [55]					WL (12) *	DWL (30 min) RCFd	RCFi *
Hudak et al., 2013 [37]		TMTB *	TMTA * DSS * UFOV * LC * MC *		WL (12)	DWL (30 min)	
Yasar et al., 2011 [38]	PVF + SVF+	TMTB *	TMTA *		WL (12) *	DWL (20 min)	
Bucur & Madden., 2010 [46]	TMTA-TMTB + SCW		DSS				
Brady et al., 2005 [39]	SVF	DSB	SC		WL (10)	DWL (5 min)	
Waldstein, Brown et al., 2005 [31]		DSB TMTB *	TMTA * MSM *	SCW	HM VM *	DHM (30 min) DVM * (30 min)	
Waldstein & Katzel, 2004 [36]		DSB VMSB *	MSM *		HM VM	DHM (30 min) DVM (30 min)	
Saxby et al., 2003 [48]	TMTA + TMTB + SVF + PVF *	NWM *	RT *		WL (12) + DWL + RWL + DRWL *		
Harrington et al., 2000 [49]					CDR	CDR	

* Significant difference; P: Significant positive correlation: u: Significant u-shaped correlation: + Compound measures; VF: Verbal Fluence; PVF: Phonological verbal fluency; SVF: Semantic verbal fluency; WCST: Wisconsin card test; LNS: Letter Number Sequence; LCT: letter cancellation test; TO: Temporal orientation; S7: Subtract 7 by 7; IC: Image copy; TMTB: Trail Making Test B; TMTA: Trail Making Test A; DSB: Digit Span Backwards; DSF: Digit Span Forward; NWM: Numeric work memory; Ar: Arithmetic; AF: Alpha Span; VMSB: Visual memory Span Backwards; DSS: Digit-symbol substitution; MSM: Manual speed motor; RT: Reaction time in retention and memory tests; SC: Stroop color; SCW: Stroop color-word; SDMT: Symbol Digit Modalities Test; LC: Letter comparison; WL: Wordlist (number of words); DWL: Delayed wordlist (waiting time); HM: History memory; DHM: Delayed history memory; VM: Visual memory; DVM: Delayed visual memory; EBMT: East Boston Memory Test; CDR: Cognitive Drug Research Computerized Assessment; RCFi: Rey complex figure test- immediate; RCFd: Rey Complex Figure Test-delayed; RM: Raven Matrices; MR: Mathematical Reasoning; BD: Blok Desing; PC: Picture Completion; PA: Picture arrangement; OA: Object Assembly; CLOX: Clok Drawing Test; EPS: Every problem solving test.

## Data Availability

The data presented in this study are available on request from the corresponding author.

## Appendix A. PRISMA (Systematic Revision Report Elements and Meta-Analysis Protocols) Verification List, 2009

**Section/Topic**	**#**	**Checklist Item**	**Reported on Page #**	
**TITLE**				
Title	1	Identify the report as a systematic review, meta-analysis, or both.	1	
**ABSTRACT**				
Structured summary	2	Provide a structured summary including, as applicable: background; objectives; data sources; eligibility study criteria, participants, interventions; study appraisal and synthesis methods; results; limitations; conclusions and key findings implications; systematic review registration number.	1	
**INTRODUCTION**				
Rationale	3	Describe the rationale for the review within the context of what is already known.	2	
Objectives	4	Provide an explicit statement of questions being addressed with reference to participants, interventions, comparisons, outcomes, and study design (PICOS).	3	
**METHODS**				
Protocol and registration	5	Indicate if a reviewed protocol exists, if and where it can be accessed (e.g., Web address); and, if available, provide registration information including registration number.	NA	
Eligibility criteria	6	Specify study characteristics (e.g., PICOS, follow-up length) and report characteristics (e.g., years considered, language, publication status) used as criteria for eligibility, giving rationale.	3	
Information sources	7	Describe all information sources (e.g., databases with coverage dates, contact study authors to identify additional studies) in the search and date last searched.	3	
Search	8	Present full electronic search strategy for at least one database, including any limits used, such that it could be repeated.	3	
Study selection	9	State the process for selecting studies (i.e., screening, eligibility, included in systematic review, and, if applicable, included in the meta-analysis).	3	
Data collection process	10	Describe method of data extraction from reports (e.g., piloted forms, independently, in duplicate) and any processes for obtaining and confirming data from investigators.	3	
Data items	11	List and define all variables for which data were sought (e.g., PICOS, funding sources) and any assumptions and simplifications made.	3	
Risk of bias in individual studies	12	Describe methods used for assessing risk of bias of individual studies (including specification of whether this was done at the study or outcome level), and how this information is to be used in any data synthesis.	3	
Summary measures	13	State the principal summary measures (e.g., risk ratio, difference in means).	3	
Synthesis of results	14	Describe the handling data methods and study combining results; if done, including consistency measures (e.g., I^2^) for each meta-analysis.	3	
Risk of bias across studies	15	Specify any assessment of risk of bias that may affect the cumulative evidence (e.g., publication bias, selective reporting within studies).	NA	
Additional analyses	16	Describe methods of additional analyses (e.g., sensitivity or subgroup analyses, meta-regression); if done, indicating which were pre-specified.	NA	
**RESULTS**				
Study selection	17	Give numbers of screened studies, assessed for eligibility, included in the review, with reasons for exclusions at each stage, ideally with a flow diagram.	4	
Study characteristics	18	For each study, present characteristics for which data were extracted (e.g., study size, PICOS, follow-up period) and provide the citations.	4	
Risk of bias within studies	19	Present data on risk of bias of each study and, if available, any outcome level assessment (see item 12).	18	
Results of individual studies	20	For all outcomes considered (benefits or disadvantages), present, for each study: (a) simple summary data for each intervention group (b) effect estimates and confidence intervals, ideally with a forest plot.	4–12	
Synthesis of results	21	Present results of each meta-analysis done, including confidence intervals and consistency measures.	4–12	
Risk of bias across studies	22	Present any assessment of risk of bias results across studies (see Item 15).	18	
Additional analysis	23	Give results of additional analyses if done (e.g., sensitivity or subgroup analyses, meta-regression [see Item 16]).	NA	
**DISCUSSION**				
Summary of evidence	24	Summarize the main findings including the strength of evidence for each main outcome; consider their relevance to key groups (e.g., healthcare providers, users, and policy makers).	14	
Limitations	25	Discuss limitations at study and outcome level (e.g., risk of bias), and at review-level (e.g., incomplete retrieval of identified research, reporting bias).	14	
Conclusions	26	Provide a general interpretation of the results in the context of other evidence, and implications for future research.	14	
**FUNDING**				
Funding	27	Describe funding sources for the systematic review and other support (e.g., supply of data); role of funders for the systematic review.	15	

From: Moher D, Liberati A, Tetzlaff J, Altman DG, The PRISMA Group (2009). Preferred Reporting Items for Systematic Reviews and Meta-Analyses: The PRISMA Statement. PLoS Med 6(7): e1000097. doi:10.1371/journal.pmed1000097.

## Appendix B. The Newcastle-Ottawa Scale (NOS) for Assessing the Quality of Case-Control Studies in Meta-Analyses

**Study**	**Selection**			**Comparability**	**Exposure**				**Total Quality Score**
	**In the Case Definition**	**Representativeness of the Cases**	**Selection of Controls**	**Definition of Controls**	**Comparability of Cases and Controls**	**Ascertainment of Exposure**	**Same Method of Ascertainment for Cases and Controls**	**Non-Respose Rate**	
Wei et al., 2018 [42]	1	1	1	1	1	1	1	0	7
Yeung et al.,2017 [52]	1	1	1	1	1	1	1	1	8
Nguyen et al., 2017 [55]	1	0	1	1	1	1	1	1	7
Hudak et al., 2013 [37]	0	1	1	1	0	1	1	1	6
Yasar et al., 2011 [38]	1	1	1	1	1	1	1	1	8
Bucur & Madden., 2010 [46]	1	0	1	1	1	1	1	1	7
Brady et al., 2005 [39]	1	1	1	1	1	1	1	1	8
Waldstein, Brown et al., 2005 [31]	1	0	1	1	1	1	1	1	7
Waldstein & Katzel, 2004 [36]	1	0	1	1	1	1	1	1	7
Saxby et al., 2003 [48]	1	1	1	1	1	1	1	1	7
Harrington et al., 2000 [49]	1	1	1	1	1	1	1	1	7
